# How similar are responses to background motion and target displacements?

**DOI:** 10.1007/s00221-022-06436-1

**Published:** 2022-08-16

**Authors:** Emily M. Crowe, Patou Vellekoop, Chermaine van Meteren, Jeroen B. J. Smeets, Eli Brenner

**Affiliations:** Department of Human Movement Sciences, Institute of Brain and Behavior Amsterdam, Amsterdam Movement Sciences, Vrije Universiteit Amsterdam, 1081 BT Amsterdam, The Netherlands

**Keywords:** Online control, Interception, Perturbation, Endpoint

## Abstract

When making a goal-directed movement towards a target, our hand follows abrupt background motion. This response resembles that of a shift in the target’s position. Does background motion simply change the position towards which the movement is guided? If so, the response to background motion should resemble the response to a target displacement. To find out whether this is the case, we ran two exploratory studies where we asked participants to hit a moving target at a specified moment. At various times during the hand’s movement, the background could move briefly at one of several speeds, and for various durations. The response to abrupt background motion was larger when the background moved later in the movement and when the background moved faster, in line with known responses to target displacements. The response to a second epoch of background motion was smaller than it would have been if there had been no first epoch, in contrast to responses to multiple target displacements. If the background was already moving before the target appeared, the hand even moved in the opposite direction. Thus, the response to background motion and that to a target displacement are clearly not identical, but they do share several features.

## Introduction

There is abundant evidence that people use many aspects of visual information to guide their goal-directed movements. This is often studied by changing visual information when the movement has already started. This method has be used to show that people respond quickly to changes in several attributes of a target including its position (e.g. Georgopoulos et al. 1981; Goodale et al. 1986; Pélisson et al. 1986; Prablanc and Martin 1992; Brenner and Smeets 1997; Day and Lyon 2000; Gritsenko et al. 2009), motion (e.g. Brenner et al. 1998), and orientation (e.g. Brenner and Smeets 2009; Desmurget et al. 1996). The response is more vigorous if a change in target position is larger (Brenner and Smeets 1997; Veerman et al. 2008) and if there is less time to adjust to the change (Liu and Todorov 2007; Oostwoud Wijdenes et al. 2011; Franklin and Wolpert 2008; Brenner et al. 2022). Such responses help people reach their goals.

People also respond to motion near the target: whenever obstacles (Aivar et al. 2008) or even irrelevant background items (Brenner and Smeets 1997, 2015; Gomi et al. 2006; Saijo et al. 2005; Zhang et al. 2019) start to move the hand deviates from its path in the direction of such motion. This is a very robust effect that is sometimes called the manual following response. Although several explanations have been proposed (e.g. Gomi 2008; Grierson and Elliot 2009; Zhang et al. 2019; Crowe et al. 2021), it remains somewhat unclear what mechanism underlies this response. The reported latency of such responses is often slightly longer than that of responses to target displacements, but that could be due to the longer time needed to detect motion than to update a position.

To successfully hit a target that is suddenly displaced or starts moving, the planned endpoint of the movement must be updated according to the change in position. When the background starts moving but the position of the target does not change, the manual following response is counterproductive because the hand moves away from the target. This futile response to background motion in the vicinity of the planned endpoint might arise because any motion onset near that position shifts the planned movement endpoint in the direction of the background motion (Brenner and Smeets 1997). The ongoing movement is then guided towards this shifted endpoint (Crowe et al. 2021). If so, the responses to perturbing the target and moving the background are both the consequence of a shift in the planned endpoint of one’s action such that we would expect them to be similarly modulated by various factors. We, therefore, conducted an exploratory study to investigate to what extent the response to background motion resembles the response to a shift in target position (or motion) by assessing several aspects of the response.

First, we investigated whether the response to background motion is time-dependent. Perturbing the target of a reaching movement and the visually perceived location of a participant’s hand are commonly used manipulations to gain insight into the mechanisms underlying the online correction of visually guided movements (e.g. Hesse and Franz 2009; Pelisson et al. 1986; Prablanc and Martin 1992; Desmurget and Grafton 2000; Saunders and Knill 2003, 2005). Studies using the aforementioned manipulations have shown that corrections to an ongoing movement when the target of that movement is displaced are more vigorous when there is less time to adjust the movement (Liu and Todorov 2007; Oostwoud Wijdenes et al. 2011; Franklin and Wolpert 2008; Brenner et al. 2022). Zhang et al. (2018) found some evidence that this also applies to the response to background motion: participants with longer movement times had less vigorous responses. We, therefore, expect more vigorous responses to background motion if it occurs later in an ongoing movement.

Second, we assessed whether the response to background motion is proportional to the size of the background’s displacement. Unsurprisingly, the response to a target perturbation is proportional to the magnitude of the shift in position (Brenner and Smeets 1997; Veerman et al. 2008) because one’s goal is to hit the target. Faster background motion leads to a larger displacement of that background, so it is expected to elicit a larger response of the hand (as demonstrated by Zhang et al. 2018). Another way to modify the displacement of the background is by varying the duration of background motion. Longer background motion results in a larger total displacement of the background. Moreover, it might influence the planned movement endpoint for a longer time and thereby result in a larger overall response of the hand.

Third, we were interested in whether the response to short intervals of background motion are independent of each other. When a target undergoes multiple perturbations within a single movement, the response to the second perturbation is no different from a response to a single perturbation at that time (Oostwoud Wijdenes et al. 2011). This is the case irrespective of whether the second perturbation cancels or doubles the initial one: each independent response is proportional to each independent target displacement. In a situation in which the movement does not have to shift back to reach the target, we also expect that the response to background motion at a certain time will be independent of any preceding intervals of background motion.

Thus far, we have considered the duration of background motion and multiple independent episodes of background motion. It remains to be seen what the response of the hand looks like for a continuously moving background. Studies that report a manual following response use a single transient episode of motion that starts abruptly when, or shortly after, the target is presented. This following response is in the opposite direction to many other motion effects. For example, in the Duncker illusion, an object appears to be moving in the opposite direction to a continuously moving background (Duncker 1929). In line with this, several studies have presented findings that can best be explained by the target appearing to have moved further in the opposite direction than the continuously moving background (Smeets and Brenner 1995; Soechting et al. 2001; Brouwer et al. 2003; de Dieuleveult et al. 2018), in accordance with the relative motion between the background and target being attributed to the target (Duncker 1929; Zivotofsky 2004). What determines whether the hand moves in the same or opposite direction to background motion?

Our goal was to investigate to what extent the response to background motion resembles the response to a target perturbation. We selected a target whereby responding to background motion was not counterproductive. Specifically, we used a horizontal bar extending for the full width of the screen, together with lateral background motion. In this way, a lateral response of the hand does not jeopardize the success of the interception. We manipulated the onset-time, speed, duration, and continuity of the background motion to assess how each of these characteristics influence the magnitude of the hand’s response. We evaluate whether the responses resemble documented responses to target perturbations. Since the main experiment left us with some questions about both the influence of background motion before the target appears and the duration of background motion, we conducted a second experiment to assess these issues specifically.

## Methods

### Participants

Twelve participants (11 right-handed; 26 ± 4 years) volunteered to take part in Experiment 1 and 12 different participants (12 right-handed; 21 ± 3 years) took part in Experiment 2 in return for course credit. The study was part of a research programme that has been approved by the local Ethics Committee in accordance with the Declaration of Helsinki. All participants gave written informed consent and were debriefed at the end of the experiment.

### Set-up

**Fig. 1:**
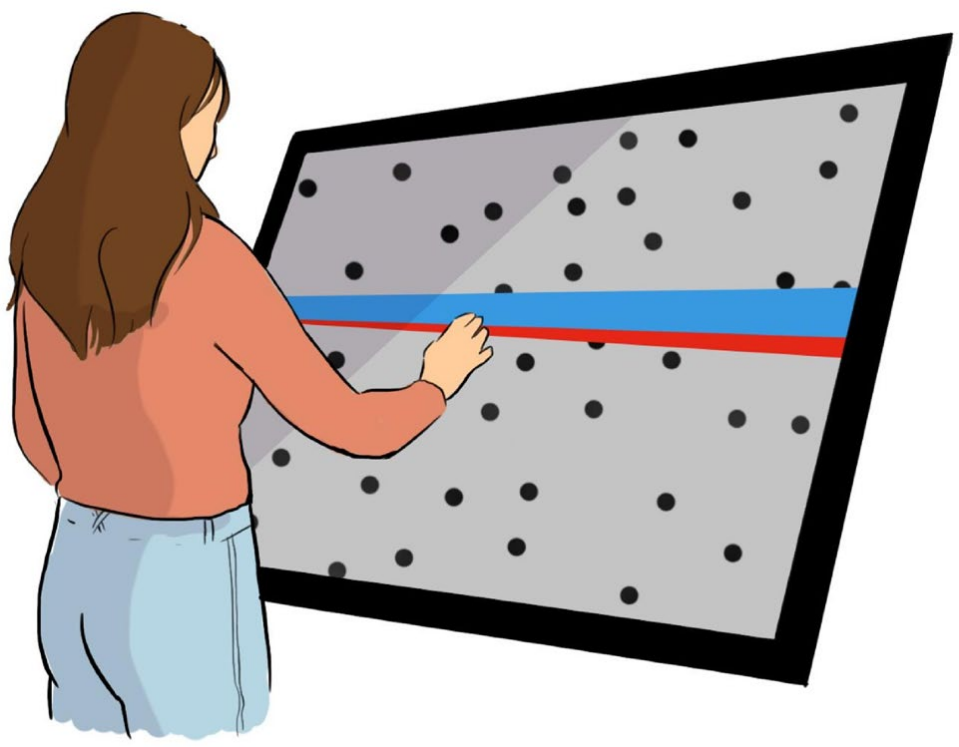
Schematic representation of the set-up. The participant stood in front of a large screen on which the stimuli were displayed. Their task was to hit the blue moving target bar when that bar was aligned with the red static bar

The experiment was conducted in a normally illuminated room. The stimuli were back-projected at 120 Hz with a resolution of 800 × 600 pixels onto a 1.25 × 1.00-m acrylic rear-projection screen (Techplex 15, Stewart Filmscreen Corporation, Torrance, California, USA) tilted backward by 30 degrees. Participants performed the task using their dominant index finger while standing in front of the screen (Fig. 1). They were not restricted in any way, so they could move as they wished. An infrared camera (Optotrak 3020, Northern Digital) that was placed at about shoulder height to the left of the screen measured the position of a marker (an infrared light emitting diode) attached to the nail of the participant’s dominant index finger at 500 Hz with sub-mm spatial resolution.

### Calibration

At the beginning of each block, the position of the marker on the fingertip was measured while the participants put their fingertip at four indicated positions on the screen. This simple four-point calibration was used to relate the position of the fingertip to the projected images, automatically correcting for the fact that the marker was attached to the nail rather than the tip of the finger.

In order to synchronize the movement data (i.e. the marker position) with the stimulus presentation, the camera also recorded the position of a second marker attached to the side of the screen. This static marker stopped emitting infrared light when a light sensor at the top left corner of the screen detected light. On each trial, we presented a flash at this position when the target was presented, when the background motion started and ended, and when the screen was hit. The briefly missing marker enabled us to synchronize the movement recording with the stimulus presentation, so we could determine the onset of perturbations and performance (e.g. whether the target was hit) at 2 ms resolution.

### Stimulus and procedure

The starting screen consisted of a green starting point (2 cm diameter) presented 20 cm below the screen centre, a red horizontal bar (115 cm × 2 cm) 10 cm above the screen centre and 250 black dots (1.4 cm diameter) randomly positioned across the grey background. To start a trial, participants placed the index finger of their dominant hand on the starting point, so they could rest whenever they wanted to by not placing their finger at the starting point. After between 500 and 700 ms, the starting point disappeared and a blue horizontal target bar (115 cm × 2 cm) appeared 30 cm above the screen centre. It immediately started moving downward at 30 cm/s. If participants lifted their finger from the starting point before the blue target appeared, the target bar did not appear and they had to place their finger back at the starting point. Participants were instructed to hit the blue moving target bar when that bar was aligned with the red static bar by lifting their finger off the screen and then tapping the screen at the correct time and location. The two bars were perfectly aligned 667 ms after the blue bar appeared. This allowed sufficient time for us to present (and manipulate) the background motion and still capture the response of the hand before the interception took place. The red bar was always at the same place and both bars covered the full width of the screen such that the emphasis was on hitting the screen at the right vertical position and time.

Participants received feedback on every trial. A tap was characterized by the acceleration of the finger being more than 50 m/s^2^ in the direction away from the screen. We interpolated the position of the target between image presentations. We considered the target to have been hit successfully if the calibrated marker position was within both the target and the static red bar at the moment of the tap. If participants successfully hit the target, the target remained at the position at which it was hit for 500 ms and a tone indicated that the hit was successful. If participants hit the target, but above or below the red static bar, the target stopped but there was no tone. If participants hit above or below the target, the target was deflected away from the finger at 100 cm/s (upward or downwards, indicative of whether the participant hit below or above the target, respectively), also remaining visible for 500 ms unless it left the screen before that. This feedback allowed the participants to keep their movements calibrated both spatially (Smeets et al. 2006) and temporally (de la Malla et al. 2014).

In addition to the target moving downwards, the background of dots always moved laterally at some moment during the trial. Different parameters of the dots’ motion were manipulated in Experiments 1 and 2, and are detailed below. In both experiments, the dots’ motion was entirely irrelevant to the participants’ interception task.

### Exploratory data analysis

Any trials that included missing data (less than 1.5% in both experiments) were excluded from subsequent analysis. All other trials were included, irrespective of performance (i.e., whether or not the target was hit). We converted the measured lateral positions of the finger into (signed) lateral velocities by direct differentiation. This was done for every 2 ms interval. For each participant, we then took the median lateral velocity of the finger for every moment separately for trials in which the background moved leftward and those where it moved rightward. We subsequently determined the (signed) *response* by subtracting the median velocity of the finger on leftward motion trials from that on rightward motion trials. Since positive laterally was to the right, a positive response is a response in the direction of background motion. We did this separately for each condition in the experiment. After determining the individual responses for each condition, mean values across participants were calculated.

To get a qualitative impression of the vigour of the response, we analyse the time-course of the responses to background motion (mean and standard error across participants). To quantify the overall influence of the perturbation, we subtracted the median movement endpoint for leftward background motion trials from that on rightward background motion trials to obtain the difference in movement endpoint for each participant in each condition. To explore how this *endpoint effect* (mean and standard error across participants) depends on the amount of background motion, we plot the endpoint effect as a function of the difference between the overall displacement of the background for leftward and rightward motion for each condition (i.e., rightward displacement—leftward displacement). To explore how this endpoint effect depends on the time with no motion at the end of the movement, we also plot the endpoint effect as a function of the time from the offset of (the last episode of) background motion until the time of a perfect hit (667 ms after target appearance).

### Experiment 1

**Fig. 2:**
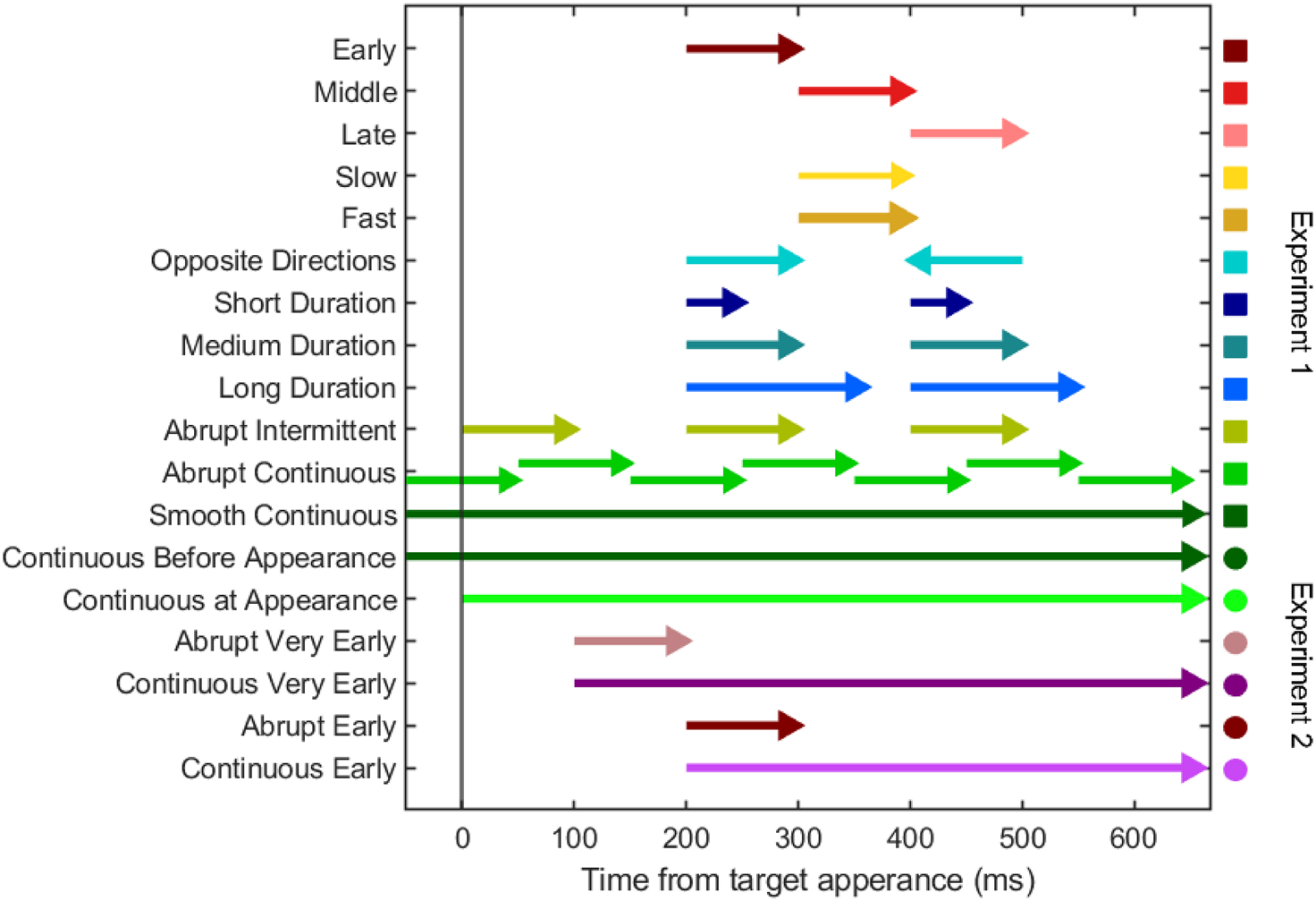
Overview of the timing of each perturbation in experiments 1 and 2. Squares indicate conditions from experiment 1; circles indicate conditions from experiment 2. The arrows indicate the presence of background motion (randomly leftward or rightward), so their length corresponds to the duration of each period with background motion. The leftward cyan arrow indicates that motion in that period was in the opposite direction than in preceding period. The thicker and thinner arrows indicate faster and slower background motion, respectively. The colour coding corresponds to the conditions and matches that in Figs. 3, 4 and 5

To investigate whether the response to background motion is time-dependent, we manipulated the timing of the onset of the background motion. To investigate whether the response is proportional to the displacement of the background, we manipulated the speed and duration of the background motion. By comparing conditions with different numbers of epochs of motion, we assessed whether the response to background motion depends on preceding epochs of background motion. This resulted in twelve experimental conditions (Fig. 2). For each of the twelve conditions, there was leftward background motion (leftward followed by rightward in the Opposite Directions condition) on 30 trials and rightward background motion (rightward followed by leftward in the Opposite Directions condition) on 30 trials. This totalled 720 trials, with all conditions randomly interleaved. The full experiment took approximately one hour. It consisted of two blocks (360 trials each) that were completed in a single visit to the lab. Participants were given the opportunity to have a break in between blocks.

In the Opposite Directions condition, the background moved sequentially in opposite directions on any given trial (indicated by the change in arrow orientation in Fig. 2), separated by an interval. In all other conditions, the background moved exclusively either rightward or leftward on any given trial. In the Fast condition the background moved at 200 cm/s and in the Slow condition it moved at 50 cm/s. In all other conditions it moved at 100 cm/s. In the Smooth Continuous condition, all the background dots continued moving laterally throughout the entire trial (from when the starting point first appeared). In the Abrupt Continuous condition, each dot moved for 100 ms and was then static for 100 ms before starting to move again. The dots moved asynchronously, so that at every frame about 10 dots started to move and 10 other dots stopped moving, with half of the dots moving at any given time throughout the entire trial, but each dot abruptly starting to move every 200 ms. In all other conditions the 250 dots always started and stopped moving at the same time.

## Results

**Fig. 3:**
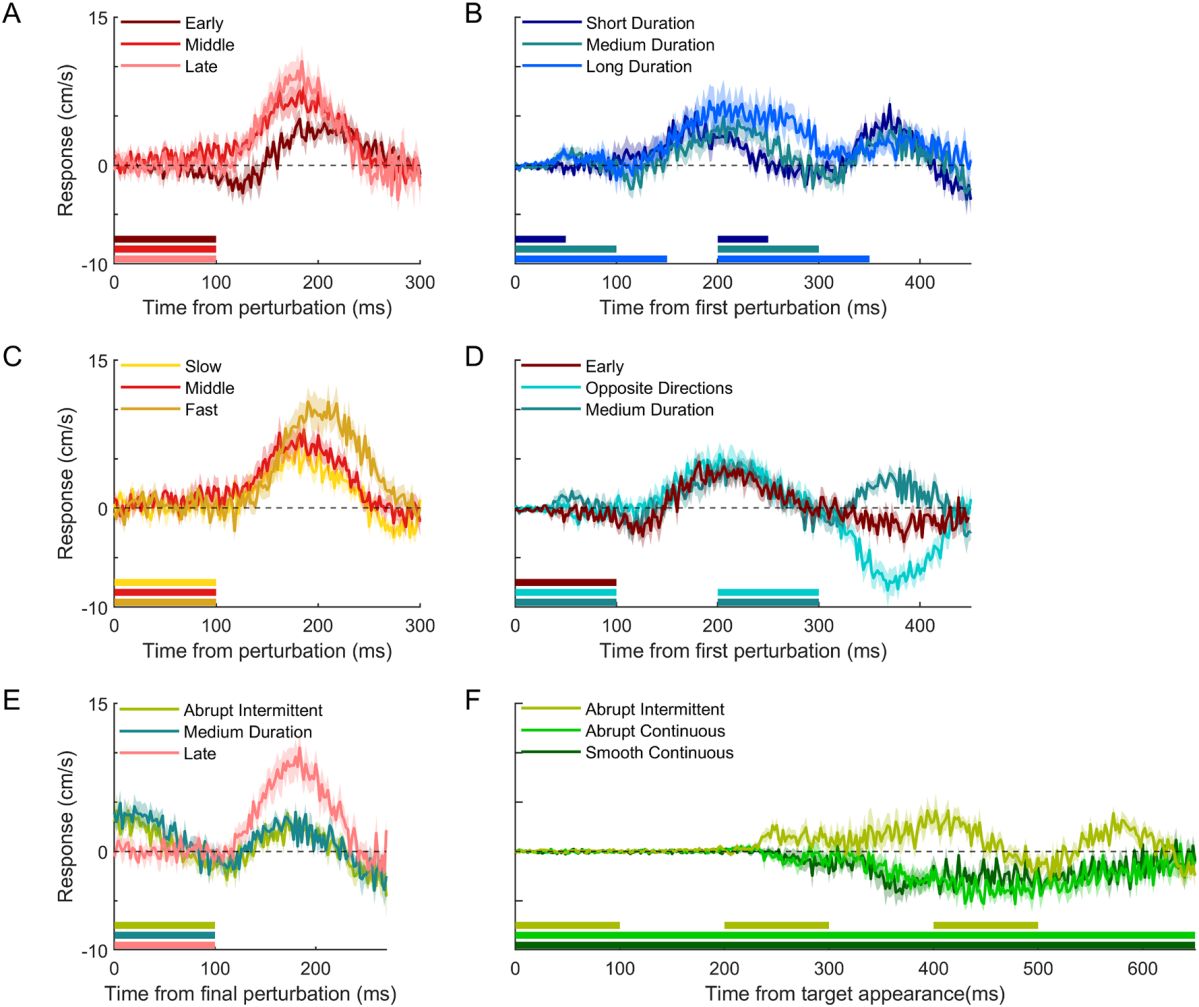
Time course of the hand’s response to background motion in all conditions of Experiment 1. Each panel shows a set of conditions (details in Fig. 2) assessing a different characteristic of background motion: **A** The effect of onset time; **B** The effect of duration; **C** The effect of speed; **D** The effect of the relative direction of a second perturbation; **E** The effect of repeat perturbations; **F** The effect of motion continuity. Each curve shows the difference between the lateral hand velocity on rightward and leftward trials as a function of time from the onset of the relevant perturbation (or target appearance in F). The curves are the means across participants; shaded regions show the corresponding standard error of the mean. A positive response is in the direction of (the initial) background motion. The coloured bars at the bottom show the timing of background motion in each condition

Participants left the starting point (moved 1 cm in the direction of the targets) 276 ± 29 ms after the target appeared (mean ± standard deviation across participants’ mean values). They hit the screen 666 ± 12 ms after the target appeared, so on average they hit the moving target when it was aligned with the static bar, but there were some individual biases. They hit both bars (successful hits) on 71 ± 10% of the 720 trials. Figure 3 shows the responses of the hand to background motion for all conditions.

The magnitude of the response is larger when the perturbation occurs later in the movement, showing that the response is time-dependent (Fig. 3A). It is also larger when the background moves longer (first peaks in Fig. 3B) or faster (Fig. 3C), showing that the response depends on the total displacement of the background. The dependence on the duration of background motion shows that the background’s displacement during some interval is relevant, rather than only the instantaneous velocity when the background starts to move. When the background moves twice in the same direction on the same trial, the response to the second motion onset is smaller, despite being later, especially for the longer duration of background motion, and therefore shorter duration with no motion (Fig. 3B). This shows that the response to the second epoch of background motion depends on the first and thus, that they are not independent of each other.

A possible reason for the response to a second motion onset being smaller than that to a first (compare the second peak of the Medium Duration and the Late conditions in Fig. 3E) is that the hand had already responded in the direction of the second perturbation by the time it takes place. Indeed, when two perturbations are applied in the same direction, the response to the second perturbation appears to be smaller than when they are applied in opposite directions (compare the medium duration and the opposite directions condition in Fig. 3D). However, this is probably at least partly the consequence of the response to the first perturbation not yet having ended, because although the second peaks in the medium duration and opposite directions conditions are clearly asymmetrical with respect to the baseline (zero), they are less asymmetrical with respect to the response to the first perturbation (the early condition consists of a single perturbation at the same time in the movement as the first perturbations in these conditions, Fig. 3D). That the response to a second motion onset is smaller than the response to a similar motion onset on its own is most evident when the responses to identical perturbations at the same time in the Medium Duration (or Abrupt Intermittent) and Late conditions are compared (Fig. 3E).

We were also interested in the response of the hand to a continuously moving background. When the whole background was moving continuously from well before the target appeared, the hand moved in the opposite direction to the background motion (negative responses in Fig. 3F), in line with results from earlier experiments with similar background motion. This response became visible when participants initiated their movement towards the target (which varied between participants, ranging from 195 to 265 ms after target appearance). The response was very similar when the whole background moved smoothly (Smooth Continuous condition) as when each background dot repeatedly moved for 100 ms and was then static for 100 ms, with dots initially starting to move at different moments so that only a selection of the background dots was moving at each instant but there was always motion in the background (Abrupt Continuous condition). When all dots started and stopped moving at the same time, even the hand’s response to the first perturbation followed the background (positive early response for the Abrupt Intermittent condition).

The two continuous conditions differed from the Abrupt Intermittent condition in two ways: the presence of motion before the target appears and the presence of motion throughout the whole movement. We propose that it must have been the presence of motion before the target appeared that made the hand move in the opposite direction because participants immediately responded in the direction of background motion in the Abrupt Intermittent condition despite the fact that, at this point in their movement, they could not know whether the background would continue to move. To confirm this, and to better assess how the duration of background motion influences the response, we conducted a second experiment.

### Experiment 2

To confirm that the direction of the response to background motion, and therefore presumably also the underlying mechanism, changes if the background is already moving before the target appears, we manipulated the moment of background motion onset. To evaluate the influence of the duration of background motion we compared responses to a 100 ms epoch of background motion early during the movement (two abrupt conditions, background motion starting at 100 or 200 ms after target appearance) with responses to continuous background motion starting at the same moment (four continuous conditions, background motion starting before, at, 100 ms after, or 200 ms after target appearance.) For each of the six conditions, there was leftward background motion on 30 trials and rightward background motion on 30 trials. This totalled 360 trials with all conditions randomly interleaved. There was a single session that was completed in a single visit to the lab and took approximately 30 min.

In all conditions the background moved at the same speed as in most conditions of experiment 1: 100 cm/s. In the continuous conditions, after the background dots first started moving laterally they continued to do so until the end of the trial. In the abrupt conditions, the background dots moved for a single epoch of 100 ms.

## Results

**Fig. 4:**
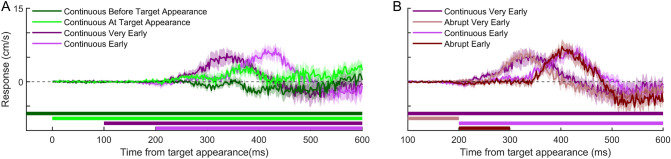
Time course of the hand’s responses in all conditions of Experiment 2. **A** How the moment at which the background starts to move influences the response. **B** How the duration of background motion influences the response. The curves are the means across participants; shaded regions show the corresponding standard error of the mean. A positive response is in the direction of background motion. The coloured bars at the bottom show the timing of background motion in each condition

Participants left the starting point (moved 1 cm in the direction of the target) 289 ± 46 ms after the target appeared (mean ± standard deviation across participants’ mean values). They hit the screen 670 ± 9 ms after the target appeared, so on average they hit the moving target when it was aligned with the static bar. They hit both bars (successful hits) on 62 ± 9% of the 360 trials. Figure 4A and B show the responses of the hand for all conditions.

We confirmed that the response is negative if background motion is present before the target appears and positive if the background motion starts at the time of the target appearance or later (Fig. 4A). Extending the duration of background motion hardly influences the response: the responses in the abrupt conditions and in the corresponding continuous conditions remain similar beyond the first 100 ms after the onset of the response.

**Fig. 5:**
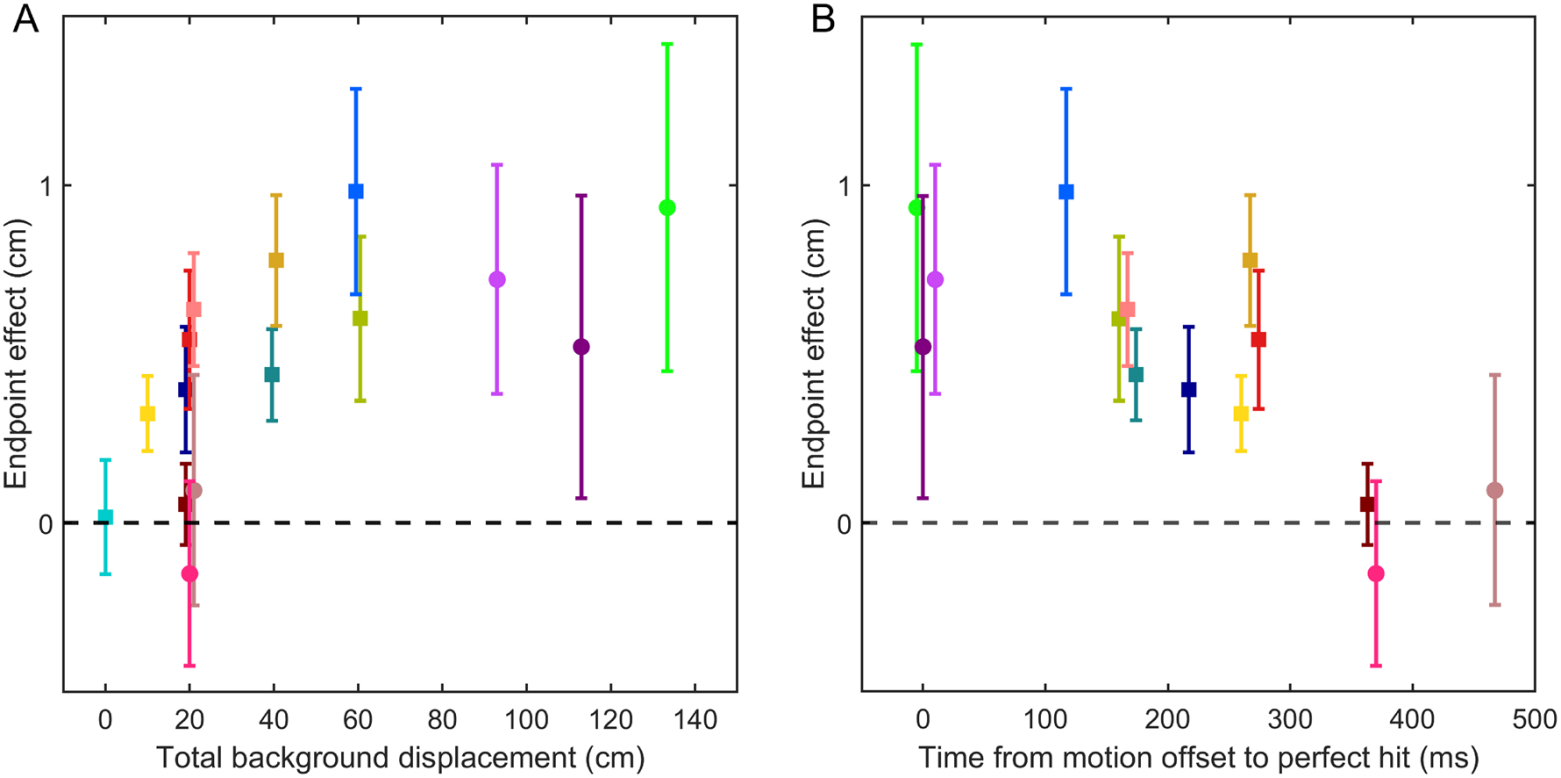
Effect of background motion on hand movement endpoints. The symbols show the means across participants; the error bars show the standard error of the mean. The colours correspond to the conditions in experiment 1 (squares) and experiment 2 (discs), see Fig. 2 for details. **A** The difference between movement endpoints for rightward and leftward background motion plotted against the difference between the total background displacements during leftward and rightward background motion. **B** The difference between movement endpoints for rightward and leftward background motion plotted against the remaining time from the last motion offset to the perfect hit (i.e. 667 ms after target appearance) for the conditions with a net background displacement

To examine how the response might correspond with a (possibly temporary) shift in the planned endpoint of one’s action, we determined the effect of background motion on the movement endpoints. Specifically, we examined how the endpoint effect depended on the total displacement of the background and on the time from background motion offset until interception. The endpoint effect increases with the total background displacement up to a displacement of about 60 cm; larger displacements have no additional effect (Fig. 5A). The endpoint effect also depends on the time between the background motion offset and the end of the movement: the shorter the time until interception, the larger the endpoint effect (Fig. 5B). This time explains why the six conditions with 20 cm total background displacement have different endpoint effects (Fig. 5A). Indeed, if the background only moves at the beginning of the trial, it does not influence the endpoint (abrupt very early, abrupt early, early, Fig. 5B). A possible interpretation is that the planned endpoint is shifted by the moving background, but slowly shifts back when the background stops moving. This would explain why the initial responses to abrupt and continuous motion are very similar (Fig. 4B) and yet the endpoint effects are quite different (Figs. 5A and B). That the planned endpoint actually moves back rather than not moving further is in line with the responses sometimes becoming negative later in the movement (Figs. 3B, D and E; 4C).

## Discussion

In this paper we investigated the extent to which the response to background motion resembles the response to target displacements. Since both responses could be interpreted as the consequence of guiding the hand to an updated planned endpoint, we expected them to be modulated by similar factors. Motion that occurred later in the movement resulted in a larger response of the hand, showing that the response to background motion is time-dependent, in line with the response to target perturbations (Brenner et al. 2022; Liu and Todorov 2007; Oostwoud Wijdenes et al. 2011). The response of the hand was also larger for fast background motion than for slow background motion, and lasted longer when the background moved for a longer period of time, suggesting that the response to background motion is proportional to the size of the perturbation, like the response to target perturbations (Brenner and Smeets 1997; Veerman et al. 2008). This also fits with findings that the response to a suddenly moving target is modulated by the target velocity (Brenner and Smeets 1997; Numasawa et al. 2022). However, the initial acceleration did not clearly scale with the speed of the background, it only continued to accelerate longer, which is not quite what one would expect.

The response to a second epoch of motion was not independent of the first epoch of motion, in contrast to the independent responses seen to target perturbations (Oostwoud Wijdenes et al. 2011). Moreover, when there was continuous motion from before the target appeared, participants moved in the opposite direction to the background motion. Thus, the response to background motion is similarly modulated by some factors, but clearly not identical to the response to target displacements.

Can we explain why the response to background motion is sometimes not equivalent to that to a target displacement? The answer may lie in the somewhat peculiar finding that for background motion the sign of the response is determined by the timing of the onset of the motion: the response is negative if background motion is present before the target appears and positive if the background motions starts at or after target appearance. Perhaps it is only the onset of background motion that shifts the position towards which the movement is guided in the direction of the motion, and after that all that remains is a modest influence of background motion on the judged position or motion of the target due to induced motion (Brouwer et al. 2003; de Dieuleveult et al. 2018). Importantly, we show that it is not only the instantaneous velocity at the onset of background motion that shifts the endpoint but rather the motion within a short time interval, because the duration of background motion does influence the response. Within some range, the response to background motion is proportional to the size of the perturbation. However, the shift of the endpoint is only a small fraction of the total background displacement. This is not surprising because moving the background might influence the judged movement endpoint, or even the judged target position, but the actual target position is unchanged.

Another potential factor limiting the magnitude of responses to background motion is that one may not only adjust the movement towards the judged position of the originally selected movement endpoint (which is presumably what the background motion influences) but also re-evaluate where one can best hit the target (Voudouris et al. 2013). This would presumably guide the movement back to the centre of the target after some delay. Experiment 2 showed that the continuity of the background motion does not affect the magnitude of the response: there was no clear difference between the response curves to abrupt or continuous background motion that occurred at the same time during the movement, even from 200 ms after the onset of background motion, when participants could have responded to the background having stopped moving. This shows that the planned endpoint does not immediately shift back when the background motion stops. To summarise, the response to background motion is not always equivalent to the response to a target displacement because only background motion within some time window from the onset contributes to the shift in the planned endpoint, and this shift is only temporary. The planned endpoint shifts back to the origin when there is no longer any motion in the surrounding. In contrast, for target displacements the planned endpoint simply shifts in accordance with the target displacement, so that the total change in position is accounted for as long as there is enough time to adjust the movement.

What information leads to the shift in the planned endpoint when the background moves? In general, the endpoint effect increased with total background displacement, suggesting that the shift in the planned endpoint is a certain fraction of the total background displacement. However, only a certain interval of the motion determines the shift, because the endpoint effect in conditions where the background moved continuously until the end of the movement is not substantially larger than conditions with only a transient epoch of motion (Fig. 5A). It is also clear that the timing of the background motion affects the movement endpoint. We found a larger endpoint effect when the background motion stops later during the movement (Fig. 5B). In fact, when the background stops moving early enough, there is no endpoint effect (the three early conditions in Fig. 5B). This drifting back corresponds with the responses that drop below zero from about 300 ms after the perturbation (Fig. 3). The endpoint effects show that our attempt to stop people from moving back to a specific endpoint by using a very long horizontal target was unsuccessful.

## Conclusions

Under the assumption that the response to background motion is due to a change in the position towards which the movement is guided, we expected the response to be modulated by similar factors as the response to target displacements (that evidently also shift the position towards which the movement is guided). In line with the response to target displacements, the response to background motion is time dependent: the response is larger when the background motion occurs later in the movement. There is also some evidence that the response to background motion is proportional to the speed and size of the background displacement. However, the response to multiple epochs of background motion is not independent and the sign of the response is determined by the timing of the onset of background motion. Although the response to background motion and target displacements are not identical, they do share several features. Thus, we propose that the onset of background motion temporarily shifts the position towards which movements are guided, but that this position gradually shifts back if the background stops moving.

## Data Availability

The data and analysis scripts for this study are available at: https://osf.io/kdfqx/?view_only=8555e01fb0c7403f86bd23a3b15ea0f1.
